# Using Dissipative Particle Dynamics to Model Effects of Chemical Reactions Occurring within Hydrogels

**DOI:** 10.3390/nano11102764

**Published:** 2021-10-19

**Authors:** Ya Liu, Joanna Aizenberg, Anna C. Balazs

**Affiliations:** 1 Chemical and Petroleum Engineering Department, University of Pittsburgh, Pittsburgh, PA 15261, USA; yal65@pitt.edu; 2 Harvard John A. Paulson School of Engineering and Applied Sciences, Harvard University, Cambridge, MA 02138, USA; jaiz@seas.harvard.edu; 3 Department of Chemistry and Chemical Biology, Harvard University, Cambridge, MA 02138, USA

**Keywords:** dissipative particle dynamics, hydrogel, chemical stimuli, complexation/decomplexation, hydrogel actuators

## Abstract

Computational models that reveal the structural response of polymer gels to changing, dissolved reactive chemical species would provide useful information about dynamically evolving environments. However, it remains challenging to devise one computational approach that can capture all the interconnected chemical events and responsive structural changes involved in this multi-stage, multi-component process. Here, we augment the dissipative particle dynamics (DPD) method to simulate the reaction of a gel with diffusing, dissolved chemicals to form kinetically stable complexes, which in turn cause concentration-dependent deformation of the gel. Using this model, we also examine how the addition of new chemical stimuli and subsequent reactions cause the gel to exhibit additional concentration-dependent structural changes. Through these DPD simulations, we show that the gel forms multiple latent states (not just the “on/off”) that indicate changes in the chemical composition of the fluidic environment. Hence, the gel can actuate a range of motion within the system, not just movements corresponding to the equilibrated swollen or collapsed states. Moreover, the system can be used as a sensor, since the structure of the layer effectively indicates the presence of chemical stimuli.

## 1. Introduction

Hydrogels are a key component in many stimuli-responsive systems because these gels act as a “muscle” that controllably actuates the system and thus enables its functionality. This muscle-like behavior is due to the material’s ability to reversibly swell and contract in response to variations in pH [1,2,3], humidity [4], temperature [5,6], or light [7] and thereby generate mechanical forces within the system. The mechanical forces enable the systems to perform useful work. For example, in systems where embedded microposts protrude from an underlying hydrogel layer, the stimuli-responsive behavior of the hydrogels allows the composite to function as a temperature-regulating, homeostatic device [2,8,9,10] or a platform for controllably trapping and releasing proteins in microfluidic chambers [11,12,13]. Typically, the hydrogels in these systems provide a binary “on/off” response: the gel exhibits an equilibrated change in volume and/or shape in the presence of the stimulus and returns to its initial equilibrium configuration when the stimulus is removed. To extend the functionality of the gels, researchers have recently designed a polymer network that forms a kinetically stable state, which can yield a variety of distinct non-equilibrium transformations. Hence, the gels can provide a variety of controllable dynamic responses beyond the typical “on/off” states.

Here, we develop a computational scheme to probe the structural evolution and dynamics of such gels and gels with embedded microplates, where specific changes in volume [14] can be tuned by tailoring the chemical stimuli in the solution. In effect, the layer forms a kinetically stable latent state, which undergoes further responsive interaction with the changing environment. For example, these gels can act as sensors that retain information concerning the presence of a stimulus, even after the stimulus is removed and thus indicate prior behavior in the system. Since the latter state is kinetically stable, the gel can be used to signal new information at a later point in time. In addition, the system can gradually release the stored mechanical strain, which can actuate a continuous action over a finite range of time. More generally, these studies provide design rules for harnessing kinetic phenomena to regulate shape changes in hydrogels and achieving control over systems that operate out of equilibrium.

Inspired by recent experiments [14], we model a hydrogel layer that is anchored to an underlying surface. In the experiments [14,15], a pH-sensitive polyacrylic acid hydrogel contracts when divalent cations in the solution (e.g., Cu^2+^) bind to the network. (In effect, the gel becomes increasingly hydrophobic as a molecule in the gel forms a complex with the divalent species.) The contraction can be reversed by introducing an ethylenediaminetetraacetic acid (EDTA) to the solution [14,15]. The acid has a preferential binding interaction with the divalent cations, leading to the extraction of the divalent cations from their complex with the gel. In this way, the gel returns to its initial non-collapsed (or essentially hydrophilic) state. (Notably, the binding of metal ions to a polymer network has been shown to shift the hydrophilic–hydrophobic balance in the system [16,17,18] and thus the complexation and decomplexation can reflect changes in the solvent quality with respect to the gel.)

Typically, polyelectrolyte effects in electrically charged hydrogels lead to changes in the swelling of the gel with variations in pH and the ionic strength in the solvent. In our study, we limit ourselves to considering a specific scenario that qualitatively mimics the behavior of the experimental system and can be achieved by adding or removing cross-linkers. Then, the osmotic pressure in the gel could be calculated using the Flory–Huggins equation, where the interaction parameters are chosen to match the experimental data on the collapsed and swollen states. This approach cannot be used to model the variety of behaviors evident in electrically charged hydrogels. Nonetheless, the model described below captures the range of essential interactions in the system: the gel responds to two chemical stimuli that occur separately in time and space, and each of these interactions involves diffusion of the molecules, complexation, and deformation of the gel.

To capture the complexity of these interactions, we augment the DPD approach [19,20,21] to simulate the kinetic behavior considered here (as discussed below). The DPD is a particle-based simulation technique where chemically reactive species are modeled on a coarse-grained, mesoscale (rather than atomistic) level. The species are depicted by a bead, which interacts with other beads to capture the salient chemical interactions. In previous studies, we successfully augmented the DPD approach to explicitly incorporate the temperature dependence of the polymer−solvent interactions in chemically cross-linked gels [22], allowing us to realistically model gels characterized by specific values of the polymer−solvent interaction parameter. We validated this model by accurately reproducing the volume phase transition of poly(N-isopropylacrylamide) (PNIPAAm) as a function of temperature [22,23]. We also developed DPD schemes to model atom transfer radical polymerization (ATRP) and free radical polymerization (FRP) and again found good agreement with experiments on the comparable systems [22,23,24].

Modeling the chemo-responsive, dynamically tunable gel (such as considered in ref. 14) requires further modification of the DPD approach. Namely, the model must capture the following interconnected dynamic events: (1) the reaction of diffusing chemicals with specific units anchored in the gel and the subsequent formation of a stable complex; (2) the accompanying concentration-dependent deformation of the gel; (3) the decomplexation within the gel in the presence of a second stimulus and the release of the bound species into the surrounding solution; and (4) the dynamic, concentration-dependent shape changes of the deformed gel. A salient feature of our newly augmented DPD model is that the physical property of a reactive bead changes once it is bound to the gel. In particular, the hydrophilicity of these bound beads is decreased relative to unbound species. This feature allows us to model the experimentally observed contraction of the hydrogel due to complexation. In addition, the relative hydrophilicity of the gel beads increases with the decomplexation, and the gel can return to the initial hydrophilic state. We further use this model to design microscopic plates that are embedded in the gels and encompass flexible hinges, which allow these plates to respond to the complexation/decomplexation occurring within the gel.

Using this augmented DPD model, we analyze changes in the volume of the gel as a function of the number density of chemical stimuli in the solution. The latter data are challenging to obtain through experimental studies. In addition, we can visualize the inhomogeneity in the structure of the gel and ascribe the inhomogeneity to molecular and nanoscale-scale interactions within the system. Namely, as discussed below, the basic unit of length in our simulations is approximately 1 nm and hence, we can capture phenomena occurring at that length scale. We also show that the cooperative interactions between the responsive gel and the embedded plates yields a chemically sensitive valve, which opens or closes depending on chemical changes in the solution. Below, we first describe the approach we used to obtain these results, which are detailed in the subsequent section.

## 2. Methodology

**Figure 1:**
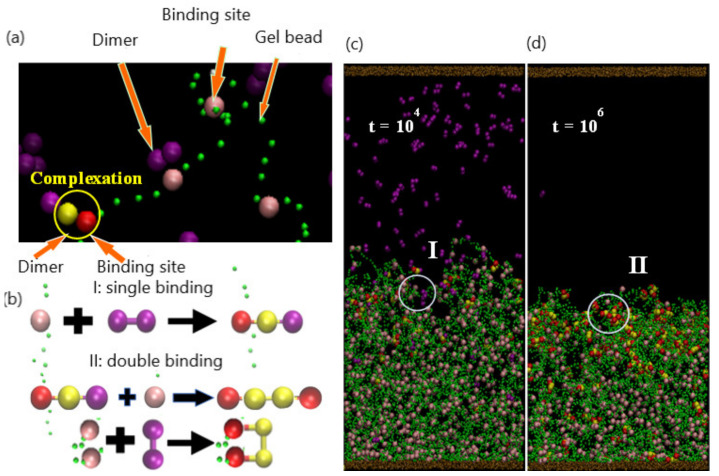
(**a**). Particle compositions of the system: dimer (purple), gel-binding site (pink), gel bead (green), and formed complexation group including a dimer bead (yellow) and a binding site (red). Beads sizes are tuned for better visualization. (**b**). Schematics for single binding (I) and double binding (II) between dimer and binding site. Double binding can be evolved from single binding or direct binding. (**c**,**d**). Snapshots of system (*N*_bind_ = 792, *N*_dimer_ = 150) at early swelling stage (*t* = 10^4^) and late collapsing stage (*t* = 10^6^). Single and double bindings are observed in I and II, separately.

As illustrated in Figure 1, the system encompasses a hydrogel (green beads) that is anchored to a substrate (brown beads) and immersed in a host solution, which is bound by the top and bottom walls of the simulation box. The hydrogel is a physically cross-linked tetra-functional network with a diamond-like topology, which is then allowed to equilibrate at the specified temperature [22]. Some of the beads are identified as specific chemical entities, indicating the species used in the experimental studies and to facilitate the ensuing discussion (as illustrated in Table 1). The gel contains chemically active binding sites—for example, acrylates (pink beads) that can form stable tetra-coordinated complexes with divalent copper (purple beads) in the solution (Figure 1a). The two purple beads can bind to two pink beads on two different strands; in the latter case, the binding introduces new chemical cross-links within the network. After complexation, the beads cannot form additional bonds and hence become inactive (red beads). The divalent copper is modeled by two interconnected beads, as detailed further below. (The latter beads are depicted as larger than the green units simply for clarity; all beads in the simulation are the same size.) This general behavior could be achieved with other types of neutral dimer cross-linkers that bind in a two-step process. We first describe the general aspects of the DPD model, allowing us to introduce and define common factors that control the dynamics of the system. Then, we discuss the specific modifications we made to the model to simulate the system in Figure 1.

Each bead *i* in the system experiences a force$\;f_{i}$, and the motion of bead i is governed by Newton’s equation of motion,$\;mdv_{i}/dt=f_{i}$. Here, **f***_i_* is the sum of three pairwise additive forces: $f_{i}\left(t\right)=\sum_{j}\left(F_{ij}^{C}+F_{ij}^{D}+F_{ij}^{R}\right)$, where the sum is over all beads j within a certain cutoff radius $r_{c}$ from bead i. The three forces are the conservative force $F_{ij}^{C}$, drag or dissipative force $F_{ij}^{D}$, and random force $F_{ij}^{R}$. We describe each pairwise force below.

The conservative force is a soft, repulsive force given by $F_{ij}^{C}=a_{ij}\left(1-r_{ij}\right){\hat{r}}_{ij}$, where $a_{ij}$ measures the maximum repulsion between beads i and j, $r_{ij}=\left|r_{i}-r_{j}\right|/r_{c}$, and ${\hat{r}}_{ij}=\left(r_{i}-r_{j}\right)/\left|r_{i}-r_{j}\right|$. While this soft-core force leads to a degree of overlap between neighboring beads, this choice for the force permits the use of larger time steps than those typically used in MD simulations [21], which commonly involve hard-core potentials (e.g., the Lennard–Jones potential). The repulsive parameter $a_{ij}$ is given in terms of $k_{B}T$. We choose room temperature as the reference value and thus $k_{B}T=1$ with T=25 °C.

The drag force is$\;F_{ij}^{D}=\gamma \omega_{D}\left(r_{ij}\right)\left({\hat{r}}_{ij}\cdot v_{ij}\right)$, where γ is a simulation parameter related to the viscosity arising from the interactions between the beads, $\omega_{D\;}$is a weight function that goes to zero at$\;r_{c}$, and$\;v_{ij}=v_{i}-v_{j}$. The random force is$\;F_{ij}^{R}=\sigma \omega_{R}\left(r_{ij}\right)\xi_{ij}{\hat{r}}_{ij}$, where $\xi_{ij}$ is a zero-mean Gaussian random variable of unit variance and $\sigma^{2}=2k_{B}T\gamma$ relates the amplitude of the noise to the friction coefficient, as specified by the fluctuation–dissipation theorem [20,21]. The value of γ is chosen to ensure relatively rapid equilibration of the system’s temperature and the numerical stability of the simulations for the specified time step [21]. Finally, we use $\omega_{D}\left(r_{ij}\right)=\omega_{R}{\left(r_{ij}\right)}^{2}={\left(1-r_{ij}\right)}^{2}$ for$\;r_{ij}<1$ [19,20,21].

Each of these three pairwise forces conserves momentum locally, and thus, the DPD simulations reproduce correct hydrodynamic behavior [19,20,21]. The velocity-Verlet algorithm is applied to integrate the equations of motion in time. We take $\;r_{c}$ as the characteristic length scale and $\;k_{B}T$ as the characteristic energy scale in our simulations. Then, the corresponding characteristic time scale is defined as$\;\tau_{\mathrm{intrinsic}}=\sqrt{mr_{c}^{2}/\;k_{B}T}$. The remaining simulation parameters are σ=3 and Δt=0.02τ, with a total bead number density of ρ=3 [21].

We take the initial configuration of the gel to be a finite-sized tetra-functional network with a diamond-like topology [22]. The semi-flexible polymer strands are modeled as a sequence of 30 DPD beads that are connected by harmonic bonds, with an interaction potential given by $\;E=\frac{1}{2}K_{\mathrm{bond}}{\left(r-r_{0}^{g}\right)}^{2}+K_{\mathrm{angle}}\left(1+\cos\theta \right)$ [23,25]. The first term in the latter expression characterizes the elastic energy with the elastic constant $K_{\mathrm{bond}}$, and the second term represents the bending energy with the rigidity parameter $K_{\mathrm{angle}}$. Here, $r_{0}^{g}$ is the equilibrium bond length and θ is the bond angle between two adjacent bonds. The bond and angle potentials of the gel are set respectively at $K_{\mathrm{bond}}=128$ and $K_{\mathrm{angle}}=4$ to prevent bond crossing and produce a polymer concentration comparable to the experimental results for this gel [26,27]. Consequently, the total force acting on each gel bead is equal to$\;f_{e}+f_{i}$, where $f_{e}=-\nabla E$ and $f_{i}$ is the DPD pairwise force as defined above.

As noted above, the divalent cations in Figure 1a, b are modeled as a dimer that is composed of two DPD beads connected through a harmonic bond. $E_{\mathrm{dimer}}=\frac{1}{2}k_{d}{\left(r-r_{0}\right)}^{2}$, where $k_{d}=256$ and $r_{0}=0.5$. The relatively high bonding strength reinforces the finite extensibility of the dimer structure compared to the gel network (where $K_{\mathrm{bond}}=128)$. In the initial setup, the dimers are randomly distributed in the bulk solution, without making any contact with gel. As the diffusion of the dimers brings them sufficiently close to the gel binding sites, the complexation reaction takes place. As a consequence, bonds are formed between the reacting components; these bonds are modeled through a harmonic spring:

(1) $$E_{bd}=\left\{\begin{array}{c} \;\frac{1}{2}k_{bd}{\left(\left|R_{b}-R_{d}\right|-r_{0}\right)}^{2},\;\left|R_{b}-R_{d}\right|<R_{\mathrm{cut}}\;\;\\ 0,\;\left|R_{b}-R_{d}\right|\geq R_{\mathrm{cut}} \end{array}\right.$$

where $R_{b}$ and $R_{d}\;$ are the respectively the coordinates of the binding site and dimer bead, and $k_{bp}=128$ is the spring constant. In the ensuing study, binding sites are uniformly distributed on the polymer gel; we consider the binding-site densities of the following values: $\rho_{b}$ = 0.037, 0.065, and 0.1, which correspond to the respective number of binding sites $N_{b}$ = 288, 504, and 792.

The complexation between the gel and copper occurs in the following manner. When a purple bead in the dimer reacts with a pink binding site (active binding site), the reacted dimer bead is converted to yellow, and the binding site is turned red (Figure 1a). In the actual, physical system, the formation of a bidentate bond typically happens through a single binding event. However, in the simulation, the formation of this bidentate bond occurs through the two steps in Figure 1a, with the formation of a single bond and subsequent formation of the second to form the double bond. This procedure is implemented for computational efficiency; the probability (and hence simulation run time) for the divalent copper to simultaneously find two binding sites within the given contact range is relatively low. Consequently, the time scale for the formation of the divalent bond in the simulation is relatively longer than that in the experiments. This relative shift in the time scale does not affect subsequent events (decomplexation), since in both the experiments and simulation, EDTA is added only after all the Cu^+2^ is bound. Notably, in the simulation, the decomplexation occurs within one step: both bonds are broken simultaneously. Unlike the complexation, the beads in the bound divalent species are sufficiently close together so that the breaking of these bonds is computationally efficient.

With respect to initial conditions, we note that the finite-sized gel network contains cross-links [22], which are beads with a connectivity of four. The network also contains dangling ends, which are located on the surface of the lattice and have a connectivity of two. Here, the gel contains 7704 beads and consists of 264 strands (segments between the cross-links), 144 cross-links, and 30 dangling ends. This network is periodic in the lateral (*x* and *y*) directions. 

The gel layer is attached via an adhesive interaction to the substrate (brown beads in Figure 1c). The effective attraction between the gel and substrate is modeled by setting the interaction parameter between the beads in the gel and bottom wall, $a_{gw}$, at $a_{gw}=a_{ps}-8$ so that the gel remains anchored to the wall in all our simulations.

The simulation box is 24.6×24.6×50 units in size and is filled with 78,778 solvent beads, maintaining the total density of the system at $\rho_{\mathrm{sys}}=3$. Eight independent simulations are carried out for $t=5\times 10^{6}$ time steps for each parameter set. (In the ensuing discussion, *t* is specified in simulation time steps, which can be related to physical units of time as discussed below.)

### Comparison of Simulation Parameters to Physical Values

We can relate the dimensionless simulation parameters to physical values in the following manner. If we assume that each solvent bead represents 10 water molecules [28,29], then a DPD solvent bead occupies a volume of $300{Å}^{3}$, since a water molecule (of mass density 1 g/cm^3^) has a volume $\approx 30{Å}^{3}$. The total bead number density in our system is $\rho_{\mathrm{sys}}=3$, and using $\rho_{\mathrm{sys}}=3r_{c}^{-3}$ and the mass density of water, we obtain the unit of length $r_{c}=0.97\;\mathrm{nm}\;$and the characteristic mass *m* = 180 Da. 

To obtain the correct characteristic time scale, we relate the collective diffusion coefficient of the polymer network in the simulations, $D_{0}^{\mathrm{sim}}=1.74\times 10^{-2}{\mathrm{nm}}^{2}/\tau_{\mathrm{DPD}}$ obtained from the swelling kinetics of the gel, to the experimental value $D_{0}^{\exp}=2\times 10^{-11}m^{2}/s$ [30]. Thus, we obtain the following physical values for the simulation parameters: $\tau_{\mathrm{DPD}}=0.87\;\mathrm{ns}$, the simulation box size is 23.9×23.9×48.5nm, and the particle diameter is 9.7 nm. Additionally, $K_{\mathrm{bond}}=128$ and $K_{\mathrm{angle}}=4$ correspond to the respective values of 0.56N/m and $4k_{B}T$ [13]. 

The DPD mutual repulsive interaction parameters between the gel beads (in green in Figure 1), the gel binding sites (in pink), and the units in the dimer (in purple) are all set equal to *a* = 25 so that the bead–bead interactions are effectively “neutral”.

## 3. Results and Discussion

### 3.1. Gel Collapse Due to the First Chemical Stimulus

A binding reaction and the formation of a complex between a bead in the dimer and the gel binding site can occur when these beads are located within the specified range of interaction. Two possible binding scenarios can occur in the formation of a complex: “single” or “double” binding, as illustrated in Figure 1b. In the case of single binding, a single dimer bead (purple) is bound to the binding site (pink) on gel; to indicate that a complex has formed, the purple bead is changed to yellow, and the pink bead is changed to red (Figure 1b(I)). In the case of double binding, the unbound purple bead in the dimer subsequently binds to an unbound pink bead (Figure 1b(II)). Alternatively, both purple beads can simultaneously form bonds with two pink beads, which can be on the same strand or two different strands (Figure 1b(II)). In all cases, dimer and binding sites that have formed a complex are respectively colored in yellow and red. 

As noted in the Section 2, the interaction energy among all gel, dimer, and binding site beads is equal to *a* = 25; the interaction energy among the solvent, green gel bead, and an unbound reactive bead (in purple or pink) is also equal to *a* = 25. On the other hand, once a dimer bead or a gel binding site forms a bond (now yellow or red), the interaction energy between the solvent and the bound beads becomes $a_{cs}=50$. Hence, the aqueous solution is a strongly unfavorable solvent for the beads that have formed a complex; in other words, the interaction between the solution and bound beads becomes hydrophobic. The value we chose for *a*_cs_ is comparable to effective repulsive parameters [31,32] extracted from a first-principles computational method, i.e., the “surface site interaction points” (SSIP) model. Here, the large $a_{cs}$ value is used to capture the interactions that lead to the collapse of the gel in the comparable experiments. The latter interaction stabilizes the complex formed in the solution. (In this way, we mimic the analogous experiments [14] where the pH-sensitive polyacrylic acid hydrogel contracts when divalent cations in the solution, e.g., Cu^2+^, bind to the network.)

Snapshots taken from the simulations at different times provide a useful means of visualizing the structural transformation of the layer. Thus, the figures described below are helpful in understanding the interrelationship between the binding (or unbinding) interactions and the morphology of the gel.

#### 3.1.1. Temporal Evolution of the Network

As an initial condition, a gel with $N_{\mathrm{bind}}=792$ binding sites (uniformly distributed along each polymer strand) is equilibrated to the swollen state at T=25°C. Initially, 150 dimers are dispersed randomly in the upper portion of the solution and do not make contact with the gel. As these dimers diffuse into the interaction range of the gel binding sites, the respective beads form a bond and initiate the formation of complexes. As shown in Figure 1c, at the early stage ($t=10^{4}$ time steps), a few complexes have formed near the top of the gel layer. The complexes that have formed predominately contain one of the dimer beads (in yellow), suggesting that at early times, the bonding occurs through the “single binding” mechanism. Notably, the presence of these bound hydrophobic sites within the gel begins to induce the collapse of the network. Recall that two purple beads can bind to two pink beads on two different strands; in the latter case, the purple beads act as a cross-linker. Hence, the dimers could more generally represent a cross-linker that forms bonds through two single binding events (even though the formation of a bidentate bond typically happens through a single binding event).

With an increase in time, the singly bound dimers can access free binding sites and cross-link the gel strands through the double-binding mechanism. Concomitantly, unbound dimers can diffuse further into the gel and directly undergo double binding. The rise in the number of bound sites increases both the hydrophobicity and the degree of cross-linking in the gel; both factors contribute to the further collapse of the network. Figure 1d reveals the morphology of the shrinking gel at the stage ($t=10^{6}$) where the absence of pink beads and presence of the red and yellow beads in the top layer indicate the predominance of doubly bound beads in this upper portion of the network. Notably, there are a few free dimers remaining in the bulk solution; however, the majority of pink binding sites are still available near the bottom of the gel.

**Figure 2:**
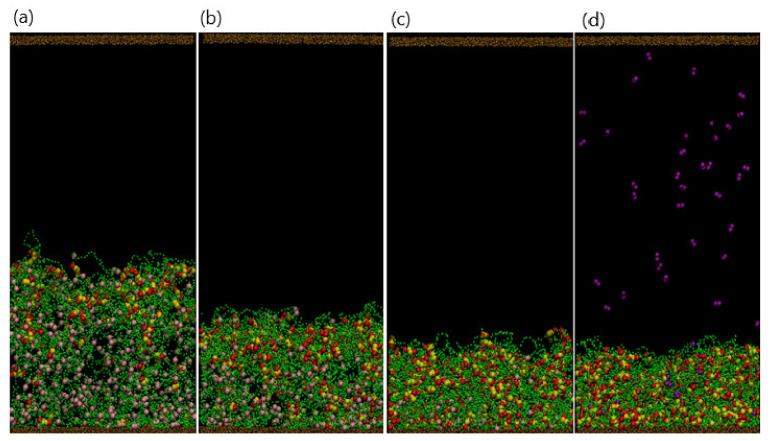
(**a**–**d**) Snapshots of system at final stage ($t=5\times 10^{6}$ for *N*_dimer_ = 150, 230, 350, and 450, respectively.

Finally, when further shrinkage is inhibited by the DPD repulsive interactions within the network and the bending rigidity of the polymer strands, the gel attains a stable, compact structure. In this collapsed configuration (Figure 2a, at $t=5\times 10^{6}$), purple dimer beads are absent from the system as all 150 dimers are bound to the gel.

#### 3.1.2. Effect of Varying the Number of Dimers in Solution 

Since the collapse of the gel is directly related to the formation of the hydrophobic complexes, varying the number of dimers, $N_{\mathrm{dimer}}$, in the solution should affect the gel’s final morphology. Figure 2 shows the final snapshots of the system (taken at $t=5\times 10^{6}$) for $N_{\mathrm{dimer}}=150$, 250, 350, and 450. Recall that the total number of binding sites is $N_{\mathrm{bind}}=792$, which limits the maximum number of doubly bound dimers in the network to 396. For $N_{\mathrm{dimer}}=150$ (Figure 2a) and 250 (Figure 2b), the complexes are concentrated near the top of the gel, leading to a dense upper layer and a more dilute lower region due to the smaller time scales involved with the binding reaction than with diffusion. In essence, the spatial distribution of complexes, as well as the gel beads, is inhomogeneous for these small values of $N_{\mathrm{dimer}}$. As $N_{\mathrm{dimer}}$ is increased, the degree of gel collapse becomes more pronounced; notably, for $N_{\mathrm{dimer}}\leq 350$, all dimer beads are bound in complexes within the gel. As $N_{\mathrm{dimer}}=350$ is close to half of $N_{\mathrm{bind}}$, the degree of gel shrinking approaches an upper limit, and the distribution of beads in the gel is more spatially uniform (Figure 2c). Finally, when the supply of dimers surpasses the gel binding capacity, for example $N_{\mathrm{dimer}}=450$ (Figure 2d), the gel reaches a maximum shrinkage of 55%. Note that at $N_{\mathrm{dimer}}=450$, a few free dimers are still localized in the solution.

**Figure 3:**
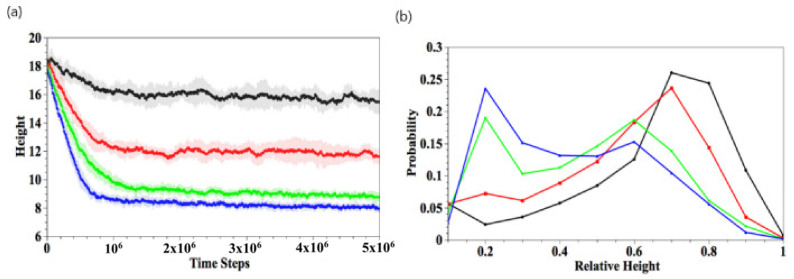
(**a**) Temporal evolutions of gel height. (**b**) Spatial distribution of bound dimers scaled by the gel height at $t=5\times 10^{6}$. Here, $N_{\mathrm{dimer}}$ = 150 (black), 250 (red), 350 (green), and 450 (blue), respectively.

To quantify the gel shrinkage induced by complexation, we calculate the temporal evolution of the gel height, $h_{\mathrm{gel}}$. The gel height is defined as the average *z*-coordinate of all the gel beads in the top layer (Figure 3a). To determine the top layer, we impose a square grid (with a grid size of one) on the bottom, lateral *xy* plane and determine the maximal height (*z*-coordinate) of gel beads in each grid; the collection of all maximal heights is the top layer of gel. The gel height is the average of the top layers in the 625 grids. Our method is consistent with the effective height determined for polymer brushes from the density profiles [33]. The black, red, green, and blue curves respectively correspond to $N_{\mathrm{dimer}}=150$, 250, 350, and 450, where 10 independent simulations are carried out for each value of $N_{\mathrm{dimer}}$. The gel height decreases rapidly in the early stage ($t<5\times 10^{5}$) for all $N_{\mathrm{dimer}}$. The absolute value of the curve’s slope, which corresponds to the shrinkage rate, increases with greater $N_{\mathrm{dimer}}$. The higher shrinkage rate at greater $N_{\mathrm{dimer}}$ is due to the higher probability that the dimers come into contact with the binding sites. In the later stages, the shrinkage rate slows down and reaches equilibrium when $t>2\times 10^{6}$. For small $N_{\mathrm{dimer}}=150$, the degree of shrinking, $d_{\mathrm{shrink}}$, is about 15% with relatively large fluctuations. For $N_{\mathrm{dimer}}=250$, 350, and 450, the respective value of $d_{\mathrm{shrink}}\approx 35\%$, 51%, and 55%, and the fluctuations in the curves become smaller.

To further characterize the final structure of the gel, we calculate the distribution of the linear density of bound dimers along the vertical direction at $t=5\times 10^{6}$, as shown in Figure 3b. The distribution *p*(s) is defined as the number of bound dimers with a z-coordinate in the range [s, s + bin size] and normalized by the total number of bound dimers. Thus, the z-coordinate ranges from 0 to gel height, which decreases as the gel shrinks. Therefore, the value of z is normalized by the gel height, thereby restricting z to lie in the range [0,1]. Figure 3b is obtained by averaging over 10 independent simulation runs. At low $N_{\mathrm{dimer}}$, the binding reaction mainly occurs on the top of the gel (rescaled height ~1), accounting for the higher density of gel in the top layer. For large $N_{\mathrm{dimer}}$, the binding interaction is more uniformly distributed. These results are consistent with the morphologies displayed in Figure 2.

**Figure 4:**
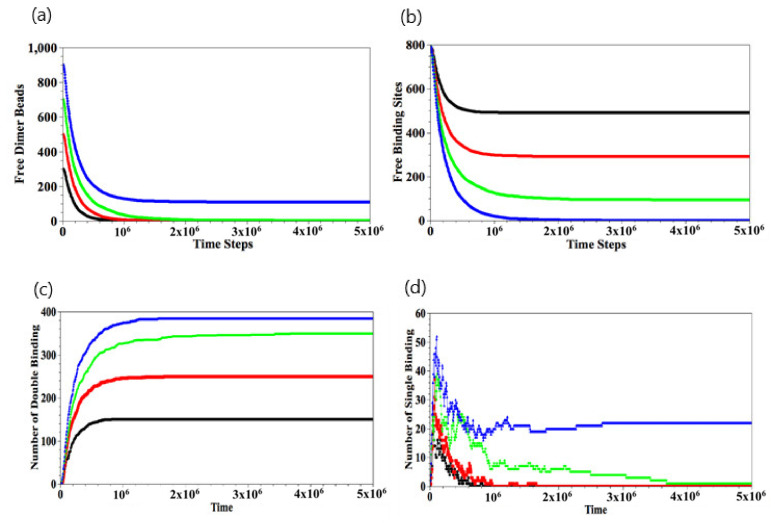
Temporal evolutions of number of free dimer beads (**a**), free binding sites (**b**), double binding (**c**), and single binding (**d**), respectively. Here, $N_{\mathrm{dimer}}$ = 150 (black), 250 (red), 350 (green), and 450 (blue). Error bars in (**a,b**) are within the width of the lines used to plot the data.

The temporal evolution of the number of free dimer beads and free binding sites is shown in Figure 4a,b, respectively. The data in Figure 4a,b are obtained by averaging over 10 independent runs, and the error bars lie within the width of the line used to plot the data. After the binding reaction has occurred for the $N_{\mathrm{dimer}}<450$ cases, the number of free dimer beads in the solution decreases and is completely depleted when $t>2\times 10^{6}$ (Figure 4a). For the latter cases, the number of available binding sites exceeds the number of $N_{\mathrm{dimer}}$, and hence, there are a finite number of free binding sites at late times (Figure 4b). As expected, the number of free binding sites decreases with an increase in $N_{\mathrm{dimer}}$. For $N_{\mathrm{dimer}}=450$, the initial number of dimers in the solution is greater than the number of available binding sites and hence, at later times, all the available binding sites in the gel are occupied, and there are a number of free dimers that remain in the solution.

When the binding between the dimers and the gel does occur, we can analyze the nature of these events by counting the number of single and double-binding interactions, as shown respectively in Figure 4c,d. For $N_{\mathrm{dimer}}<450$, the number of single-binding interactions grows sharply in the early stages and then decreases as the number of double-binding interactions grow with time (Figure 4d); eventually, the number of single-binding interactions goes to zero. On the other hand, for $N_{\mathrm{dimer}}=450$, there remains tens of single binding events since $N_{\mathrm{dimer}}>N_{\mathrm{bind}}$.

### 3.2. Reversing the Gel Collapse with a Second Chemical Stimulus

**Figure 5:**
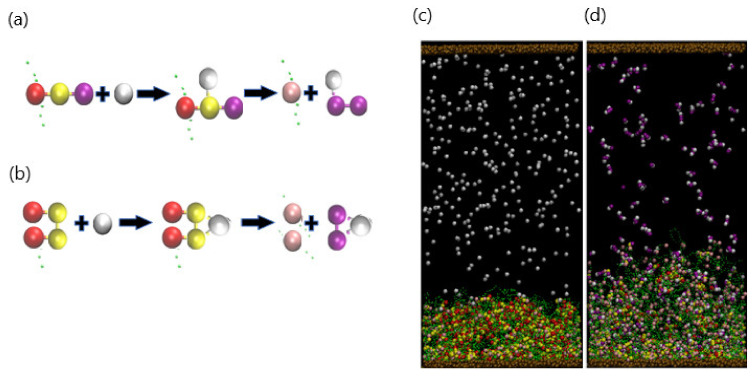
(**a**) Reactions between EDTA particle (white) and single-binding complexation. (**b**)**.** Reactions between strong-binding particle and double-binding complexation. (**c**,**d**)**.** Snapshots of the system after adding EDTA ($N_{\mathrm{EDTA}}=300$) at (**c**) $t=10^{4}$ and (**d**) $t=10^{6}$.

The collapse of the gel due to the first chemical stimulus (the dimers) can be reversed by introducing a second chemical stimulus. To analyze this behavior, we add white beads (playing the role of EDTA in the corresponding experiments) into the solution; the units in the dimers have a stronger binding affinity with these white beads than with the gel binding sites (Figure 5a). Namely, when a white bead comes sufficiently close to the bound dimer, the two bonds connecting the gel and dimer break simultaneously, and the dimer preferentially forms new bonds with the white beads. Due to these preferential binding interactions, when a white bead comes sufficiently close to the dimer, the two bonds between the gel and dimer beads are broken simultaneously. Then, the gel site reverts from a hydrophobic (red) to the initial neutral (pink) bead (see Figure 5a).

For this study, the final configuration of the collapsed gel for $N_{\mathrm{dimer}}=450$ (with the free dimers in solution removed) serves as the initial configuration for the simulations. To simplify the following descriptions, we refer to a “white bead” as EDTA and consider the cases where $N_{\mathrm{EDTA}}=100$, 200, 300, and 400 beads. (Note that additional solvent beads are added to the simulations to maintain the overall beads’ density at ρ=3.) Figure 5b shows a snapshot of the system with $N_{\mathrm{EDTA}}=200$ at $t=10^{4}$, as the EDTA diffuses toward the gel layer. At $t=10^{6}$, a significant fraction of the gel–dimer bonds have been ruptured, leading the gel to swell, and the newly formed EDTA–dimer complexes to diffuse into the bulk solution.

**Figure 6:**
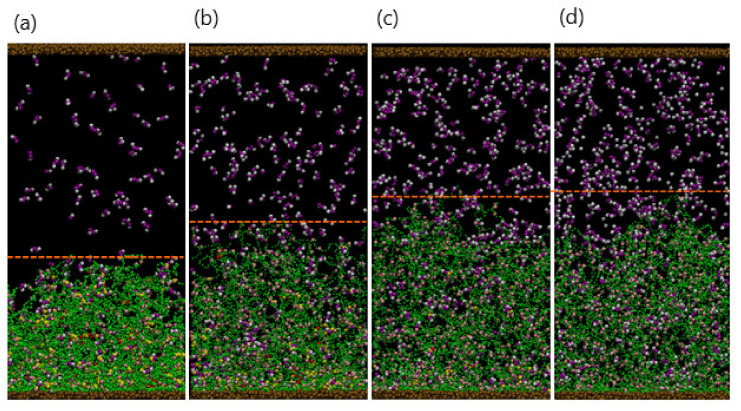
(**a**–**d**) Snapshots of system at final stage ($t=5\times 10^{6}$) for $N_{\mathrm{EDTA}}=300$, 500, 700, and 900, respectively. Dashed lines indicate the surface of the gel.

Figure 6a–d show the late stages of the system at $t=5\times 10^{6}$ for $N_{\mathrm{EDTA}}=100$, 200, 300, and 400, respectively. As seen in the figures, the extent to which the gels are swollen at late times is controlled by the $N_{\mathrm{EDTA}}$. In particular, the gel is ultimately more swollen when more *N*_EDTA_ is added to the aqueous solution; in this case, more of the bound dimers are effectively extracted from the network, making the gel more hydrophilic. The extent of swelling eventually saturates when all the dimers are released from gel when $N_{\mathrm{EDTA}}\geq 396$. To quantify the dynamics of this process, we monitor the temporal evolution of the gel height and number of the gel–dimer complexes remaining with the gel.

**Figure 7:**
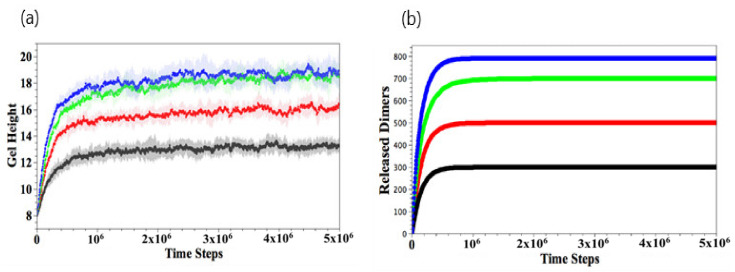
Temporal evolution of gel height (**a**) and number of released dimers (**b**) $N_{\mathrm{EDTA}}=300$ (black), 500 (red), 700 (green), and 900 (blue), respectively. The error bars in (**b**) are within the width of the lines marking the curves.

As shown in Figure 7a, the gel height increases as a function of time and increases monotonically with $N_{\mathrm{EDTA}}$. For all cases, the height reaches a plateau value at the late stages. As more dimers bind to EDTA and thus are released from gel (Figure 7b), the gel becomes less hydrophobic, leading to the reversible gel swelling.

### 3.3. Controlling the Motion of Embedded Fibers

**Figure 8:**
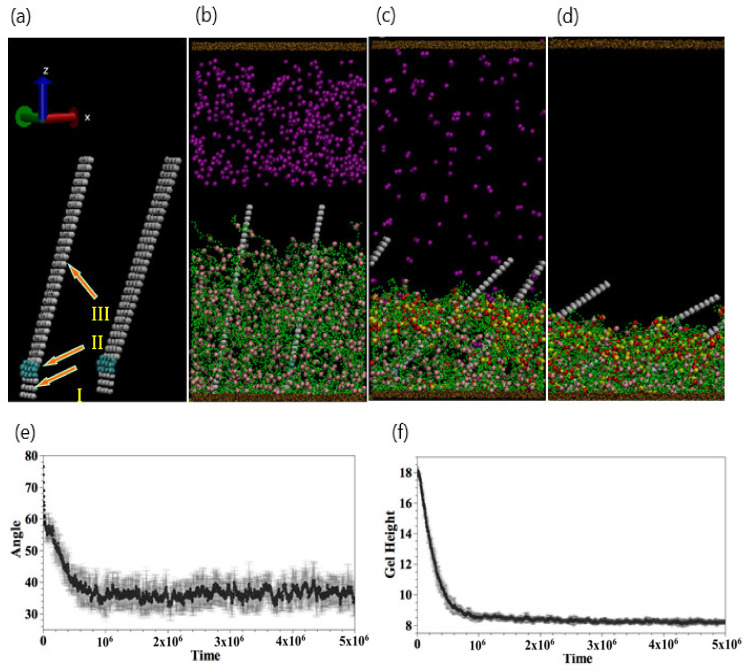
(**a**) Structural composition of plates including tethered base (I), flexible hinge (II), and rigid top (III). (**b**–**d**) Snapshots of the system after adding 700 dimmers at (**b**) t=0, (**c**) $t=2\times 10^{6}$, and (**d**) $t=5\times 10^{6}$, respectively. (**e**,**f**) are temporal evolutions of tilting angle and gel height, respectively.

This mode of regulating the gel height can be utilized to control the motion of microfibers that are bound to the gel beads and attached to the bottom wall. To illustrate this point, we introduce a DPD model for the microfibers, which are shown in Figure 8a. Each microfiber contains three subsections: Section I is the tethered end, Section II is the flexible hinge, and Section III is the rigid portion of the fiber. The structure is formed from two layers and encompasses a total of 272 DPD beads; the fiber’s respective length × width × height is equal to 4 × 2 × 34.

The elastic energy of the microfiber includes a harmonic stretching potential and a cosine harmonic bending potential, $E_{\mathrm{fiber}}=E_{s}+E_{b}$ with

(2) $$E_{s}=\frac{1}{2}K_{s}\left(\sum_{i,j}{\left(\left|r_{i}-r_{j}\right|-r_{0}\right)}^{2}+\sum_{i,j}^{\prime }{\left(\left|r_{i}-r_{j}\right|-\sqrt{2}r_{0}\right)}^{2}\right)+\frac{1}{2}K_{s}\sum_{i,j}{\left(\left|r_{i}-r_{j}\right|-r_{1}\right)}^{2}$$

and

(3) $$E_{b}=K_{\mathrm{bend}}{\left(1+\cos\theta \right)}^{2}.$$

The first two contributions in Equation (2) stand for the harmonic spring between the nearest and next-nearest neighbor beads in the same layer. The third term represents the spring connecting the two layers of beads. Here, the spring constant is $K_{s}=128$ and the equilibrium bond length is $r_{0}=0.8$. The bending potential in Equation (3) is characterized by the constant $K_{bend}=150$, and θ is the bending angle (at the hinge). The inter-layer spacing between the two bead layers of the fiber is $r_{1}=0.3$. Hence, the thickness of the microfibers is 0.3, the width is 2.4, and the height is 26.4 in dimensionless units. (As noted above, a unit corresponds to a length equal to 0.97 nm.) For these parameter values, the number density of microfibers is equal to 14. (The value of number density effectively prevents the unphysical crossing between gel strands and the fiber.).

The force acting on each bead is obtained by calculating the negative of the gradient of the energy. Additional constraints are applied to Section III (Figure 8a): the entire section moves as a rigid body. Initially, the fiber is tilted at an angle of 10° with respect to the horizontal axis; this ensures that all the fibers bend in the same direction. To model the fact that the fiber is anchored within the gel, we introduce 200 harmonic bonds connecting Section III and the gel; here, the spring constant $K_{s}=256$ and equilibrium bond length $r_{0}=0.5$. The gel beads bound to the fibers are spatially distributed around the fiber. When the gel collapses, we stipulate that the bonding force must be sufficiently high to bend the fiber, and hence, $K_{s}$ is taken to be higher than that between the beads of the microfiber. With this choice of parameters, the fibers display stable dynamic behavior, and the gel shrinks by approximately 50%, which is comparable to the experimental value.

As shown in Figure 8b, 450 dimers are introduced into the bulk solution. At $t=10^{5}$, the diffusing dimer beads come into contact with the gel, and the collapsing gel forces the fiber to bend (at the hinge). Finally, at $t=3\times 10^{6}$, the bending of the fibers reaches a limiting value. We characterize the dynamics of fibers by the mean tilt angle of two fibers. As shown in Figure 8e, the tilt angle decreases from 80° and fluctuates at about 40°. The enhanced bending of the embedded fiber is consistent with the gradual collapse of the gel, as illustrated in Figure 8f.

**Figure 9:**
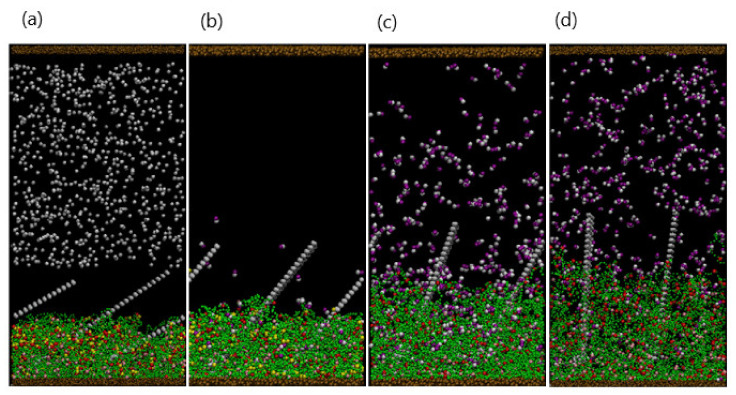
(**a**–**d**) Snapshots of the system after adding 700 EDTA particles at (**a**) t=0, (**b**) $t=2\times 10^{4}$, (**c**) $t=2\times 10^{5}$, and (**d**) $t=5\times 10^{6}$, respectively. EDTA particles are removed for better visualization in (**b**–**d**).

Notably, the bending of the fibers can be reversed by the addition of EDTA. Figure 9a–c are snapshots of the system after 400 EDTA beads are added to the collapsed gel and tilted fibers.

**Figure 10:**
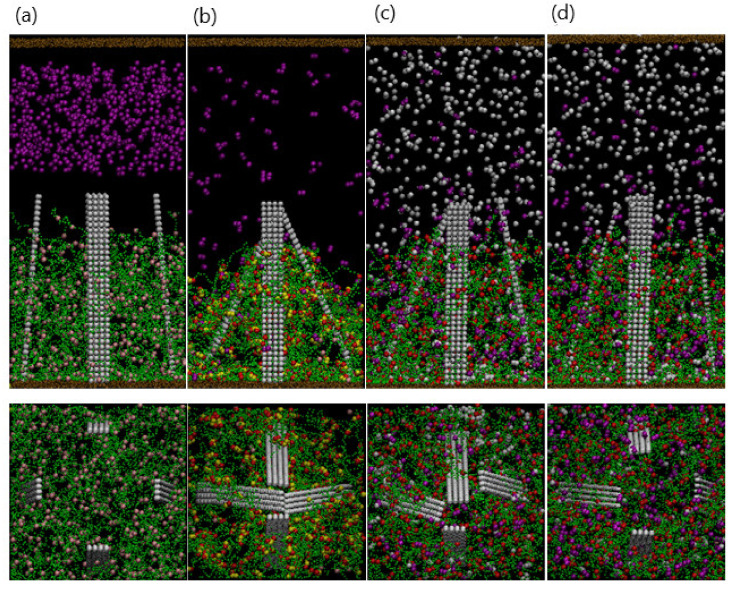
(**a**–**d**) Upper (lower) panels are top (side) view of the system with four fibers. (**a**) Addition of copper particles at t=0. (**b**) Fibers clasp at $t=2\times 10^{6}$. (**c**) Snapshot at $t=2\times 10^{5}$ after adding EDTA particles. (**d**) Fibers open at $t=2\times 10^{6}$.

Figure 10 shows a side view of the corresponding system. As the gel swells, the gel–fiber bonds drive the fibers to tilt “backward” and eventually assume a nearly vertical position (Figure 10d). The reversible, synchronized behavior of the gel and fibers can be harnessed to create chemo-responsive valves, as shown in Figure 10. In this scenario, four opposing fibers are anchored within the gel, and each fiber is tilted at a 10° angle with respect to the horizontal axis. The collapsing and swelling of the gel, and the corresponding motion of the four fibers can be controlled through the sequential addition of the dimers and EDTA. In this manner, the fibers can act as regulators that close and open in response to surrounding chemical cues. The specific fiber motion displayed in Figure 10 could provide a means of controllably trapping and releasing target particles in solution.

## 4. Conclusions

We extended the DPD approach to capture the dynamics of complexation and decomposition of a polymer gel in the presence of different chemical stimuli (e.g., polyacrylic acid gel with the sequential addition of copper ions and EDTA). In this new model, we specified the DPD bead type before and after the binding of the gel to a chemical species; we permitted different gel–solvent interactions based on the types of beads involved in the pairings. The interaction was adjusted by tuning the DPD repulsive parameter $a_{ij}$ between the solvent and complex from neutral ($a_{ij}=25$) to repulsive ($a_{ij}=50$) after the formation of the complex and vice versa if the complex is decomposed. 

Using this approach, we analyzed the behavior of hydrogel systems that can serve as finely tuned actuators as well as sensors that reveal significant information about the local environment. We specifically focused on hydrogels that are immersed in solution, and the bottom gel layer anchors the material to an underlying surface. An initial chemical stimulus was introduced through the addition of reagents when the gel was in the equilibrated, swollen state. The hydrogel reacted with these additives and sequestered certain products of the reaction within the gel layer. In turn, the sequestered molecules altered the morphology of the gel, causing it to collapse. We showed that the extent of collapse is determined by the concentration of the chemical additives in the solution. Note in the case of low dimer concentration, the polymer concentration across the height of the gel was non-uniform and leads to inhomogeneous stresses in the layer. In turn, the stresses in the sample contributed to the shape changing of the gel. We also demonstrated the reversibility of swelling/shrinking transition of the gel after introducing the second species.

This collapsed layer constitutes a kinetically stable, latent state that can be modified by the addition of a subsequent chemical stimulus. In our study, these secondary chemical additives exhibited a preferential interaction with the molecules bound within the layer and effectively extracted these species from the gel, causing it to become swollen. We also investigated the relationship between the degree of swelling and the concentration of the second additive.

The system is distinctive for a number of reasons. With variations in the concentration of additives, the layer forms a number of separate, kinetically stable configurations. Thus, the hydrogen exhibits a range of accessible states, not just the “on/off” conditions typically associated with hydrogel actuators. Hence, the functionality of gel is greatly enhanced, since this “muscle” can actuate a range of motion within the system, not just movements corresponding to the equilibrated swollen or collapsed states. Moreover, the system can be used as a sensor, since the structure (height) of the layer effectively indicates the presence of chemical stimuli. The response of the sensor can be amplified and readily read out when microscopic plates (or posts) are embedded within the layer. Then, the tilt of the microstructures can clearly indicate the presence and extent of chemical reactions with a local chemical stimulus (i.e., environmental change).

The findings from these studies are useful in highlighting regions in parameter space where the gels exhibit the most pronounced responses to the environmental changes. The results can prove useful in designing next-generation sensors, actuators, and information storage devices that operate through bioinspired chemo-mechanical energy transduction, where chemical reactions and mechanical movement are highly coupled to provide efficient forms of mechanical work.

## Figures and Tables

**Table 1:** Color map for each bead and their interaction parameter with solvent (*a*).

Color	Bead Type	Interaction with Solvent (*a*)
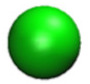	Gel	25
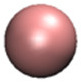	Binding site (active)	25
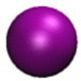	Dimer (Copper)	25
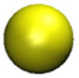	Bound Dimer	50
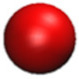	Bound Binding Site (inactive)	50
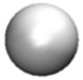	EDTA/Fiber	25
